# Editorial: The Pathogenetic Mechanisms at the Basis of Aortopathy Associated with Bicuspid Aortic Valve: Insights from “Omics”, Models of Disease and Emergent Technologies

**DOI:** 10.3389/fphys.2017.01002

**Published:** 2017-12-04

**Authors:** Amalia Forte, Alessandro Della Corte

**Affiliations:** ^1^Department of Experimental Medicine, University of Campania “L. Vanvitelli”, Naples, Italy; ^2^Department of Cardiothoracic and Respiratory Sciences, University of Campania “L. Vanvitelli”, Naples, Italy

**Keywords:** bicuspid aortic valve, aortopathy, pathogenesis, hemodynamics, genetics, epigenetics, smooth muscle cells, endothelial cells

The bicuspid aortic valve (BAV) represents the most frequent congenital cardiovascular malformation among live births, with a prevalence of 1–2% in the general population (Ward, 2000).

BAV aortopathy is characterized by a complex, multifactorial pathophysiology, involving alterations in genetics, epigenetics, hemodynamics, as well as in cellular and molecular pathways (Forte et al., 2013, 2016). All these factors contribute to BAV aortopathy with different levels of interaction and impacts in patients. BAV aortopathy is associated with a 6-9-fold increased risk of aortic complications (e.g. dissection) with respect to the general population, leading to higher morbidity and mortality (Michelena et al., 2011). Currently, the only available treatment of aortic aneurysm is surgical intervention (Calero and Illig, 2016).

In this Research Topic of *Frontiers in Vascular Physiology*, Authors with different background and expertise in the field of BAV aortopathy, examined different aspects of this disease, including the clinical, hemodynamic and biomechanic aspects, the identification of key molecular pathways involved in alterations of the phenotype of endothelial (ECs) and smooth muscle cells (SMCs) and in mechanosensing, and the identification of potential early biomarkers of aortopathy, with the aim of collecting and summarizing the most updated research findings.

It is believed that different combinations of BAV phenotypes and valvular dysfunctions lead to different alterations in wall shear stress in distinct regions (Della Corte et al., 2014; Sievers et al., 2016), which in turn can cause consequent specific molecular and histological changes. An in-depth characterization of BAV aortopathy is still missing, at least in part because of the heterogeneity described above, thus leading to limitations in the clinical approach, i.e., risk stratification criteria and standardization of surgical treatment. Fatehi Hassanabad et al. addressed in an interesting review the risk stratification of BAV patients through non-invasive hemodynamic biomarkers derived from novel imaging techniques. Among them, the *in vivo* assessment of time-resolved 3D blood velocity, using a volumetric imaging method referred to as 4D flow magnetic resonance imaging (MRI), can be utilized to accurately identify altered flow patterns secondary to BAV, even if aortic stenosis is present. As a result, 4D flow MRI revealed that aortic wall shear stress (WSS) is increased in BAV subjects independently from the degree of stenosis, and is dependent on aortic valve fusion phenotype. The study summarized by the Authors in their review demonstrates the potential utility of 4D flow MRI to identify areas with more advanced aortopathy in patients. Advanced models of risk prediction, i.e., based not only on aortic size and growth criteria, could lead to the development of individualized surgical approaches for patients with BAV and associated aortic disease.

The importance of hemodynamics in BAV aortopathy and the role of 4D flow MRI in the identification of alterations of WSS distribution in patients in relation with BAV morphotype and before the onset of aortopathy has been underlined also by a research study conducted by Piatti et al. In addition, the relevance of BAV morphotype in pulsatile flow characteristics has been investigated and confirmed *in vitro* in a study conducted on porcine tissue models of tricuspid aortic valve (TAV) and BAV using particle image velocimetry (PIV) (McNally et al.).

Of interest, Kim et al. focused their investigations not on the aorta but on the structure and mechanical function of the common carotids of BAV and TAV patients using ultrasound and velocity vector imaging (VVI), revealing differences for carotids in BAV patients and thus suggesting intrinsic vascular alterations in these subjects.

While abnormal flow patterns in the aortas of patients with BAV are increasingly recognized as important in the pathogenesis of aortic dilatation, pulmonary flow patterns in bicuspid pulmonary valves have not been investigated so far. Bicuspid pulmonary valve disease is rare and a small numbers of case reports describe concomitant pulmonary artery dilation similar to the dilation of the ascending aorta. Bissell et al. focused their research study on the 4D flow MRI assessment of the flow pattern in bicuspid *vs*. tricuspid pulmonary valves, revealing haemodynamic alterations similar to recent studies in BAV aortopathy, thus suggesting the importance of flow patterns in the pathophysiology of vessel dilation in both aortic and pulmonary bicuspid valve disease.

Along with hemodynamics, surely genetics contributes to BAV aortopathy pathogenesis, as intuitively suggested by BAV malformation being characterized by an autosomal dominant pattern of inheritance with reduced penetrance and variable expressivity. Few genes have been robustly linked to the BAV phenotype to date. In the present Research Topic, Giusti et al. reviewed the genes and *loci* proven or candidate to be associated with BAV (e.g., NOTCH1, GATA5, ACTA2 and a locus containing AXIN1 and PDIA2). Interestingly, the Authors reported also data for the gene FBN1, whose alteration is already known to be causative of Marfan syndrome (MFS), but some variants have now been associated with aortic dilation also in some BAV patients not fulfilling the clinical criteria for MFS. In addition, the impact of novel high-throughput sequencing technologies and of methods for data analysis on research and diagnosis of BAV genetic alterations has been addressed by Giusti et al. Among them, the haloplex target enrichment system has been used by Gillis et al. for targeted resequencing of 22 BAV-associated genes and for evaluation of their contribution to the etiology of BAV-associated thoracic aortic aneurysm (TAA), starting from the hypothesis that rare genetic variants in these genes may be enriched in patients presenting both BAV and TAA. The Authors identified SMAD6 as the strongest candidate susceptibility gene, showing different types of mutations in 2.5% of the large BAV/TAA cohort they analyzed. Additional considerations about the different influence of genetics and hemodynamics in the different phenotypes (root *vs*. tubular ascending) of BAV aortopathy have been proposed by Yassine et al.

Mutations in the NOTCH1 gene mentioned above are proven to be associated with non-syndromic forms of BAV. Ignatieva et al. tested in a research study the hypothesis that Notch-dependent pathways and TGF-β and BMP differentiation pathways exhibit different alterations in SMCs isolated from aortic tissue of BAV and TAV patients, with SMCs from BAV aortas more prone to osteogenic differentiation in response to Notch signal.

Currently, there are no effective strategies to prevent or limit the progression of BAV-related diseases, including aortic dilation. The development of novel therapeutic strategies requires a more detailed comprehension of the underlying molecular mechanisms. In this context, this Research Topic counts in some interesting studies focusing on molecular players whose knowledge about their role in cardiovascular pathogenesis is increasing: the non-coding RNAs (ncRNAs), a class of molecules that includes the micro-ribonucleic acids (miRNAs) and the long non-coding RNAs (lncRNAs). miRNAs are endogenously expressed, 19–23-nt-long RNAs that regulate gene expression at post-transcriptional level, mostly via base-pairing interactions that occur preferentially within the 3′ untranslated regions (UTRs) of target mRNAs. Martínez-Micaelo et al. identified a specific pattern of miRNAs associated with plasmatic endothelial microparticles (EMPs) that is specific for BAV disease, and in particular a cluster of 19 highly co-expressed miRNAs located in the 14q32 locus that could play a role as effectors of the intercellular communication carried out by EMPs in endothelial damage in BAV aortopathy. A relevant role for endothelium in the development of TAA associated with stenotic BAV has been highlighted also in a review by van de Pol et al. with particular reference to the role of communication between ECs and SMCs. miRNAs have been also the focus of the review by Alajbegovic et al., and specifically the small RNA molecules expressed by SMCs and influencing actin polymerization during the progression of aneurysm in BAV. Actin polymerization is important to maintain the contractile phenotype of SMCs through the promotion of specific gene expression *via* the transcriptional co-activator MRTF, which is translocated to the nucleus when released from monomeric actin.

lncRNAs are longer than 200 nt and, differently from miRNAs, their distinct functions are still largely unexplored. Li and Maegdefessel summarized in their review the current knowledge about the regulatory roles of lncRNAs in the fate of SMCs and in development and progression of TAA, with particular reference to HIF1α-antisense RNA 1.

Finally, Norton and Yang provided an overview of the phenotypical heterogeneity of BAV, and the associated complications and also propose a classification of BAV patients on the basis of their clinical phenotype, suggesting a conservative approach or a surgical resection depending on the involvement of aortic root in TAA.

This forum of comprehensive reviews and research studies on distinct aspects of the pathophysiology of BAV aortopathy provides both the state of the art in the knowledge on this complex disease and novel insights into its causes and consequences. The present collection of focused papers also envisions and proposes new therapeutic strategies, novel biomarkers and original risk stratification criteria, for the improvement of patient management.

## Author contributions

AF and AD reviewed all the papers accepted for publication in the Research Topic of Frontiers in Vascular Physiology and summarized them in the Editorial, together with a general commentary on the status of current knowledge in BAV aortopathy.

### Conflict of interest statement

The authors declare that the research was conducted in the absence of any commercial or financial relationships that could be construed as a potential conflict of interest.
